# The evolving role of esophageal surgery – advancements, challenges, and the path forward

**DOI:** 10.1515/iss-2025-0005

**Published:** 2025-03-03

**Authors:** Stefan P. Mönig, Mickael Chevallay

**Affiliations:** Division of Digestive Surgery, University Hospital of Geneva, Geneva, Switzerland

Historically, esophageal surgery has been associated with significant morbidity and mortality, reflecting the complexity of the procedure and the frailty of the patient population. For decades, esophagectomy has been among the highest-risk oncologic surgeries, with postoperative complications and long recovery times affecting patient outcomes. However, driven by these challenges, surgeons worldwide have continuously refined surgical techniques and perioperative management strategies, fostering a culture of collaboration and innovation that has led to substantial improvements in patient care. Advances in perioperative optimization, multidisciplinary treatment planning, minimally invasive approaches, and the centralization of care in high-volume centers have collectively contributed to a steady decline in surgical complications and improved long-term outcomes.

Despite this progress, esophageal surgery continues to evolve. One major shift in recent years has been the growing emphasis on individualized treatment strategies, challenging the notion that all patients require immediate surgical intervention. The Dutch SANO study, for example, is investigating an “active surveillance” approach for patients achieving a clinical complete response (cCR) after neoadjuvant chemoradiotherapy (nCRT). By reserving esophagectomy for cases of residual or recurrent disease, this strategy seeks to maintain oncologic control while minimizing the risks associated with surgery.

At the same time, the role of minimally invasive surgery, particularly robotic-assisted techniques, is expanding. Complex upper gastrointestinal procedures, such as the repair of voluminous hiatal hernias, have benefited from robotic platforms, which offer enhanced visualization, greater dexterity, and improved surgeon ergonomics. Given the technical challenges associated with large hernias – such as the need for precise mediastinal dissection and durable crural repair – robotic surgery is increasingly viewed as a valuable tool.

Beyond localized disease, the concept of oligometastatic esophageal and gastroesophageal junction (GEJ) cancer is reshaping treatment paradigms. Historically, metastatic disease was treated exclusively with systemic therapy, with surgery playing no role beyond palliation. However, growing evidence suggests that in select patients with limited metastatic burden and favorable tumor biology, aggressive local treatment – whether through metastasectomy, stereotactic body radiation therapy (SBRT), or a combination of modalities – may confer a survival benefit. The German FLOT-5 (RENAISSANCE) trial is actively investigating this approach, evaluating whether curative-intent resection following induction chemotherapy can improve outcomes in oligometastatic disease.

Looking ahead, several areas of esophageal surgery demand further innovation. Perioperative care, while significantly improved, must continue to evolve with enhanced recovery protocols, improved patient selection, and better management of complications. The integration of artificial intelligence and precision oncology into treatment planning may further refine decision-making, allowing for more personalized surgical strategies. Additionally, long-term functional outcomes – particularly related to dysphagia, reflux, and quality of life – should be prioritized alongside oncologic success.

The future of esophageal cancer treatment is moving away from rigid protocols toward more flexible, patient-centered approaches. As our understanding of tumor biology grows and surgical techniques improve, the role of surgery must be continually re-evaluated. Surgeons must lead this progress, embracing innovation while ensuring that each intervention is carefully tailored to provide the best outcomes with the least risk. The challenge ahead is clear: to refine techniques, adapt to new evidence, and drive innovation so that esophageal surgery continues to improve survival and quality of life in the era of precision medicine.

